# Global trends in travel-related antimicrobial resistance: a systematic review, 2020–2024

**DOI:** 10.1093/inthealth/ihaf071

**Published:** 2025-10-29

**Authors:** Georgina Tetteh-Ocloo, Alex Odoom, Nicholas T K D Dayie, Eric S Donkor

**Affiliations:** Department of Medical Microbiology, University of Ghana Medical School, P. O. Box KB 4236, Korle Bu, Accra, Ghana; Department of Medical Microbiology, University of Ghana Medical School, P. O. Box KB 4236, Korle Bu, Accra, Ghana; Department of Medical Microbiology, University of Ghana Medical School, P. O. Box KB 4236, Korle Bu, Accra, Ghana; Department of Medical Microbiology, University of Ghana Medical School, P. O. Box KB 4236, Korle Bu, Accra, Ghana

**Keywords:** antimicrobial resistance, global surveillance, international travel, resistant bacteria, systematic review, travel-related

## Abstract

Antimicrobial resistance (AMR) is a growing global health threat, with international travel playing a key role in the spread of resistant bacteria. This systematic review examines trends in travel-associated AMR from 2020 to 2024. A search of PubMed, Scopus and Web of Science identified 10 studies involving 359 AMR isolates. Using the Newcastle–Ottawa scale, the study quality was assessed and findings were synthesised to identify patterns in prevalence, diversity and geographic spread. Results revealed a consistent rise in travel-associated AMR, particularly from regions such as Southeast Asia and Africa, which acted as major sources of diverse resistant pathogens. These include extended spectrum beta-lactamase-producing *Escherichia coli*, multidrug-resistant (MDR) *Corynebacterium diphtheriae* and colistin-resistant Enterobacterales. The number of MDR strains increased over time, making up 15.3% of cases by 2024. Healthcare exposure during travel emerged as a significant risk factor. Overall, the prevalence and diversity of AMR bacteria linked to travel have risen steadily, highlighting the urgent need for global cooperation. Enhanced surveillance, antimicrobial stewardship, infection control measures and international collaboration are essential to curb the spread of these dangerous pathogens.

## Introduction

Antimicrobial resistance (AMR) has emerged as a significant global public health concern in recent decades and poses a serious threat to the effective prevention and treatment of various infectious diseases. The WHO has declared AMR a ‘global health security threat’ and has called for urgent action to address this pressing issue.^1^ One of the key drivers of the global AMR crisis is the interconnected nature of the modern world, where the rapid movement of people, goods and pathogens across borders has facilitated the spread of resistant microorganisms. International travel has been identified as a significant contributing factor to the dissemination of antimicrobial-resistant pathogens.^2,3^

Given the increasing frequency and volume of global travel, the threat of travel-related AMR is particularly concerning. According to the United Nations World Tourism Organisation, international tourist arrivals reached 1.5 billion in 2019, a significant increase from previous decades.^4^ This unprecedented level of international mobility has created ample opportunities for the cross-border transmission of antimicrobial-resistant microorganisms, further intensifying the global AMR crisis. The spread of AMR through travel-related pathways poses a significant public health challenge, with far-reaching consequences. Resistant pathogens acquired during travel can be introduced into new geographical regions, leading to the emergence and dissemination of resistant strains in local populations.^5^ This can compromise the effectiveness of antimicrobial treatments, making infections more difficult to manage and increasing the risk of adverse health outcomes. Moreover, the burden of travel-related AMR extends beyond individual health. The economic implications are also substantial, as the treatment of resistant infections often requires more costly and complex interventions, leading to increased healthcare expenditures and lost productivity.^6^ Global health security implications are also concerning, as the unchecked spread of resistant microorganisms can undermine the ability of countries to respond effectively to infectious disease outbreaks and pandemics.^7^

The scale and impact of travel-related AMR are well documented in the literature. A systematic review published in 2020 found that the prevalence of antimicrobial-resistant gut colonisation among international travellers ranged from 20 to 80%, with higher rates observed in individuals travelling to low- and middle-income countries.^8^ Another study reported that travellers returning from the Indian subcontinent had an increased risk of acquiring extended spectrum beta-lactamase (ESBL)-producing Enterobacteriaceae compared with those who did not travel.^9^ The consequences of travel-related AMR are also evident in the data. A study conducted in the Netherlands found that nearly one-third of all ESBL-producing *Escherichia coli* infections in the country were attributable to international travel.^10^ Similarly, a report from the European Centre for Disease Prevention and Control (ECDC) estimated that travel-associated infections contribute to approximately 10–20% of all carbapenem-resistant Enterobacteriaceae (CRE) cases in the European Union.^11^

Addressing this public health challenge is urgent because of the increasing prevalence and global spread of antimicrobial-resistant pathogens associated with travel. In 2021, a systematic review provided insights into the impact of travel on the dissemination of AMR worldwide.^5^ Given the growing recognition of the importance of this issue, there is a need for a comprehensive and up-to-date synthesis of the current literature on travel-related AMR. Therefore, we synthesised existing evidence on the relationship between travel and the acquisition and transmission of antimicrobial-resistant pathogens, focusing on the period from 2020 to 2024.

## Methodology

This systematic review was conducted in accordance with the Preferred Reporting Items for Systematic Reviews and Meta-Analyses (PRISMA) guidelines.^12^ The PRISMA framework provided a standardised approach to the planning, conduct and reporting of the systematic review, ensuring transparency and methodological rigour. The review proposal was preregistered on the Open Science Framework and can be accessed at https://doi.org/10.17605/OSF.IO/4HG5M.

### Search strategy

A comprehensive search of electronic databases was performed to identify relevant studies published from January 2020 to December 2024. The search was conducted on 23 December 2024. The databases searched were PubMed, Scopus and Web of Science.

The search strategy involved a combination of keywords and Medical Subject Headings (MeSH) terms related to travel, AMR and their various synonyms. The search terms included but were not limited to (‘travel’ OR ‘pilgrim*’ OR ‘Hajj’ OR ‘Hadj’ OR ‘Haj’ OR ‘Olympic’ OR ‘overseas student’ OR ‘international student’ OR ‘immigrant’ OR ‘world cup’ OR ‘mass gathering’ OR ‘crowding’ OR ‘tourism’ OR ‘travel medicine’ OR ‘holiday’) AND (‘drug resistance’ OR ‘antimicrobial resistan*’) (Table S8).

The search strategy was designed to capture a broad range of study designs, including observational studies (e.g. cohort, case-control, cross-sectional), interventional studies and modelling studies. Additionally, the reference lists of the included studies and relevant review articles were manually searched to identify any additional eligible studies.

### Study selection

Following the database search, all retrieved records were imported into Rayyan^13^ for screening and selection. The study selection process involved two independent reviewers who assessed the eligibility of the studies based on the predefined inclusion and exclusion criteria.

Inclusion criteria:

- Studies that examined the association between travel and the acquisition or transmission of antimicrobial-resistant bacteria, viruses, fungi or other microorganisms.
- Studies conducted in any geographical region or country.
- Studies published in peer-reviewed journals from January 2020 to December 2024.

Exclusion criteria:

- Studies not published in English.
- Conference abstracts, editorials, commentaries and review articles without original data.

Any discrepancies between the reviewers during the study selection process were resolved through discussion or consultation with a third reviewer, if necessary.

### Data extraction

A standardised data extraction form was developed to collect relevant information from the included studies. The data extraction was performed by two independent reviewers, and the following information was collected:

- Study characteristics: author(s), publication year, study design, study setting and study population.
- Travel-related factors: destination, purpose of travel, and travel-associated exposure (e.g. healthcare, food and water, social activities).
- AMR-related data: type of resistant microorganisms, prevalence or incidence of acquisition, transmission patterns and risk factors.

### Quality assessment

The methodological quality of the included observational studies in this systematic review was assessed using the Newcastle–Ottawa scale (NOS).^14^ The NOS is a validated tool for the quality assessment of non-randomised studies, evaluating them across three domains: selection of the study groups, comparability of the groups and ascertainment of the exposure or outcome of interest. Each domain was scored on a scale of 0–4 (selection), 1 (comparability) and 0–3 (outcome/exposure), resulting in a maximum total score of 8 stars. Studies that scored ≥7 stars were generally considered to be of high quality, while those scoring 5–6 stars were deemed to be of moderate quality and those scoring <5 stars were considered to be of low quality. The NOS assessment was conducted independently by two reviewers, and any discrepancies were resolved through discussion or consultation with a third reviewer.

### Data analysis and synthesis

Given the anticipated heterogeneity in the study design, population and outcome measures, a narrative synthesis of the findings was conducted. The geographical distribution of the included studies was visualised using a world map that highlighted the regions and countries where research on travel-related AMR was conducted. This mapping facilitated the identification of hotspots and geographic disparities in the available evidence. To complement the visual representations, detailed tables were constructed to provide a comprehensive summary of the included studies, key findings and quality assessment scores. These tables serve as a reference point for the narrative synthesis.

### Ethical considerations

This study was a systematic review of the published literature; thus, no direct ethical approval was required. The review adhered to ethical principles, such as respecting the confidentiality and integrity of the data reported in the included studies.

## Results

### Search results and articles

The initial literature search yielded 2076 potentially relevant articles from Scopus (*n*=558), PubMed (*n*=1180) and Web of Science (*n*=338). An additional 10 articles were identified through manual search techniques on Google Scholar and reference lists, resulting in 2086 articles. After removing 1140 duplicate records, 946 articles were screened based on their titles and abstracts. During this screening, 843 articles were excluded, leaving 103 articles that were then subjected to full-text screening.

During the full-text screening, 93 articles were excluded for the following reasons:

- Non-human studies
- Studies that did not address travel and AMR
- Reviews, editorial commentary and correspondence
- Lack of travel history information
- Full-text articles not available
- No information about the antimicrobials tested.

**Figure 1.**
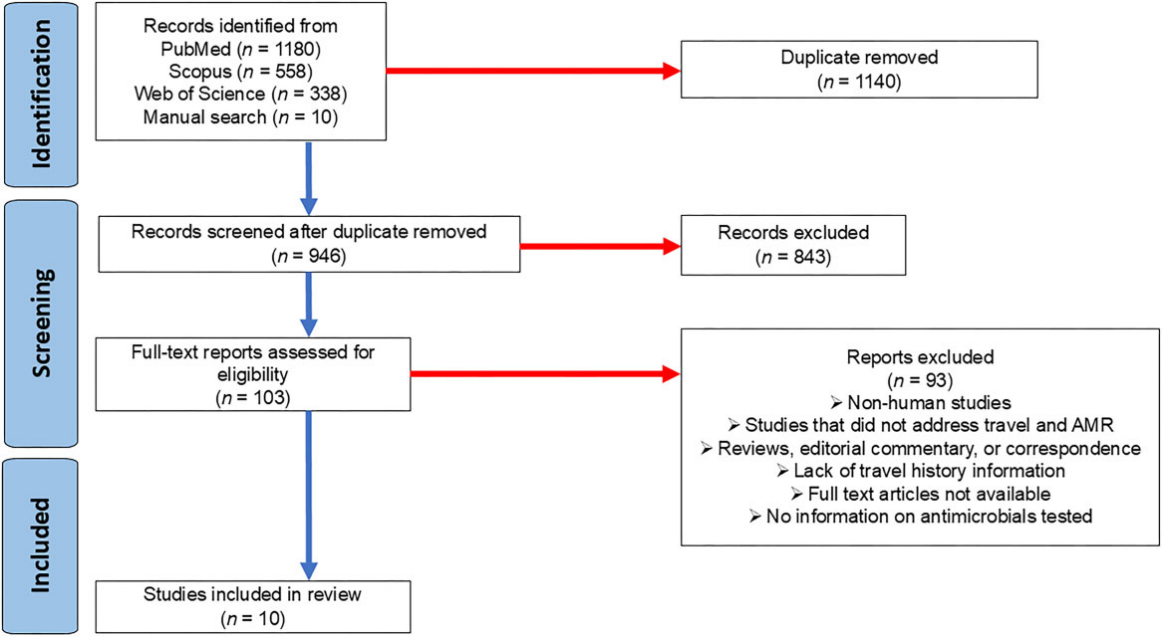
The study selection process.

After the full-text screening, 10 articles^15–24^ were deemed eligible and included for the final data extraction and synthesis (Figure 1). All ten articles were associated with AMR in bacteria.

### Characteristics of the included studies

The analysis of the 10 included studies revealed detailed information on the number of travel-related AMR isolates documented over the 2020–2024 period (Table S1). The overall NOS scores for the included studies ranged from 6 to 8 stars, with the majority (n=8, 80%) of the studies scoring 7–8 stars (Table S2). This suggests that the methodological quality of the included studies was generally high, providing a reliable foundation for the synthesis and interpretation of the findings. The isolates were categorised based on their geographic origin and AMR profiles. Overall, 359 travel-associated AMR isolates were reported across the studies (Table S5).

### Originating and destination regions of travel-associated AMR

Data from the included studies indicated that Southeast Asia and Africa were the predominant sources of travel-related AMR bacteria. The Southeast Asian region accounted for 58 AMR isolates across five studies. The specific locations within Southeast Asia that were identified as hotspots included Indonesia (34 isolates), India (26), Thailand (4) and Nepal (4).

The African continent was the origin of 70 AMR isolates, as reported in six studies. The island of Zanzibar was the most commonly cited location, with 60 AMR isolates. The other African countries mentioned were Guinea (3 isolates), Mali (3), Senegal (2), Nigeria (1) and the Central African Republic (1). An additional 147 AMR isolates were reported from unspecified or multiple regions, as documented in five studies (Table S3).

**Figure 2.**
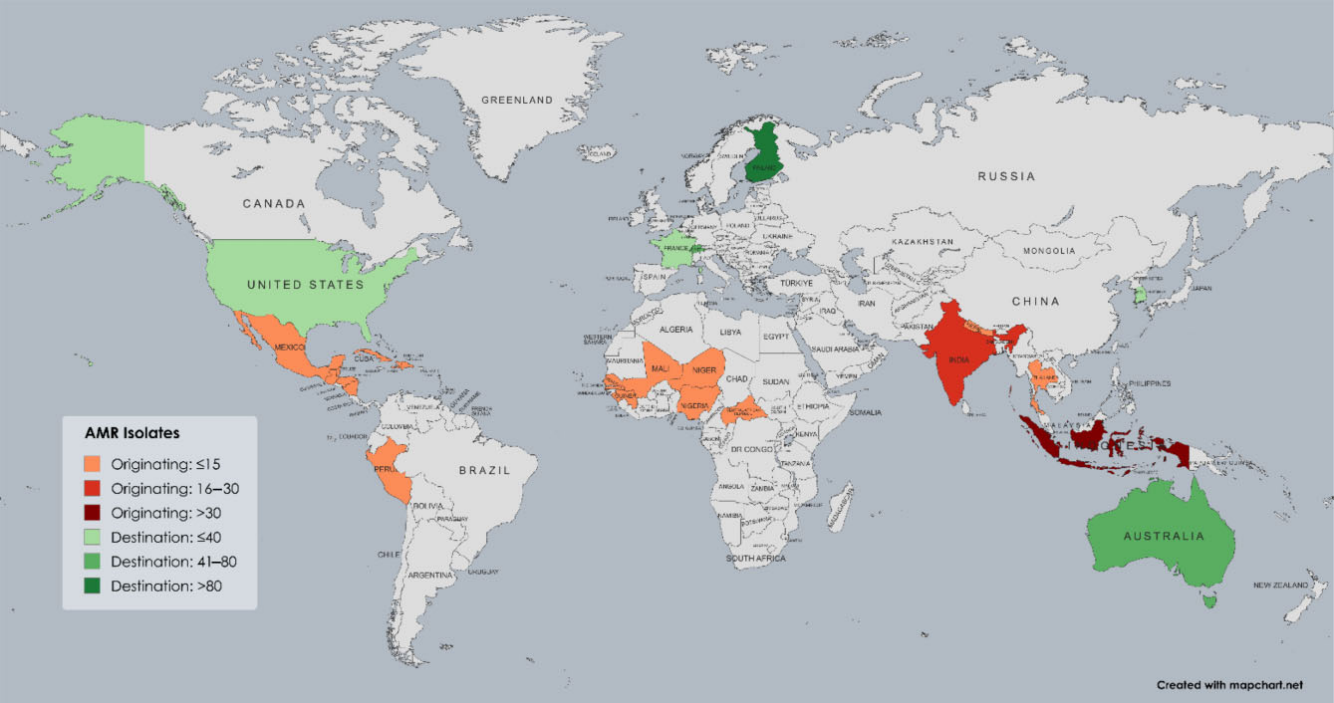
Originating and destination hotspots of travel-associated AMR. AMR: antimicrobial resistance.

The main regions of travel-associated AMR were Europe, Oceania, East Asia and North America. Europe, particularly France (38 isolates), Switzerland (60) and Finland (109), was the destination for 207 AMR isolates across five studies. The Oceania region, predominantly Australia, received 68 AMR isolates, as reported in two studies. In North America, the USA was the destination for 25 AMR isolates, as documented in one study. In East Asia, Hong Kong (60 isolates) and South Korea (8) were the destinations for 68 AMR isolates, as reported in two studies (Figure 2, Table S4).

### Resistant pathogens and antimicrobials

**Figure 3.**
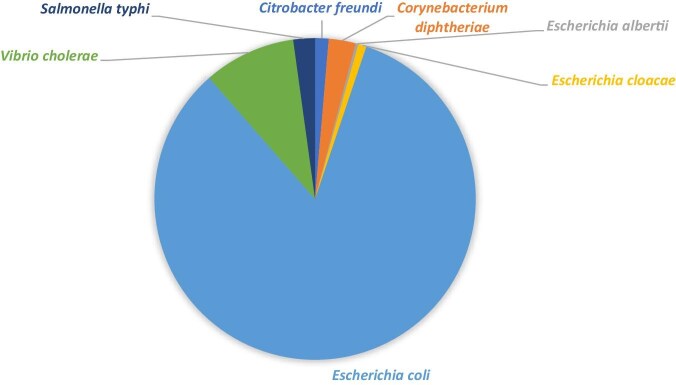
Travel-associated AMR bacteria. AMR: antimicrobial resistance.

The most commonly documented travel-associated resistant pathogens were *E. coli* (306 isolates across 7 studies), *Vibrio cholerae* (34 isolates in 1 study) and *Corynebacterium diphtheriae* (10 isolates in 1 study) (Figure 3, Table S5). Specifically, these pathogens exhibited resistance across different classes, such as 237 isolates for cephalosporins, 220 isolates for sulphonamides and trimethoprim, 345 isolates for quinolones, 171 isolates for tetracyclines, 167 isolates for penicillins, 76 isolates for macrolides and linasamides, 55 isolates for amphenicol and 107 isolates for other antimicrobials, including nitrogenfurantoin and colistin (Table S6, Table S7).

Notably, these studies reported the emergence of multidrug-resistant (MDR) strains, highlighting the increasing complexity of managing these infections. The acquisition of resistance to colistin, a last-resort antibiotic, was also documented among Enterobacterales isolates from travellers returning from Southeast Asia and Peru.

### Movements and trends of travel-related AMR bacteria

#### Temporal trends of AMR isolates

The analysis of the 10 studies included in this review offers valuable insights into the temporal trends of travel-related AMR bacteria over time. Published from 2020 to 2024, these studies provide a comprehensive examination of AMR trends during this period. In 2020, travel-associated AMR isolates were not detected. In 2021, AMR isolates began to be identified, primarily from Asia and Europe, with the most common resistance profiles observed against quinolones, sulphonamides/trimethoprim and aminoglycosides. By 2022, AMR isolates were documented, mainly from Southeast Asia, showing resistance to beta-lactams, quinolones and sulphonamides/trimethoprim. Resistance levels increased by 34% compared with the 2021 baseline. In 2023, a shift in geographic origin was observed, with AMR isolates predominantly coming from Africa and Europe, exhibiting resistance to beta-lactams, tetracyclines and aminoglycosides. Notably, colistin resistance emerged among Enterobacterales isolated from travellers returning from Southeast Asia and Peru. A sharp drop was observed in some regions (e.g. Southeast Asia), with data indicating a 0% detection rate for many drug classes, representing a 100% decrease compared with 2022. Finally, in 2024, AMR isolates surged again, with the majority originating from Southeast Asia and demonstrating resistance to beta-lactams, macrolides, quinolones and sulphonamides/trimethoprim. This marked the highest level of AMR detection across all years, with a dramatic rebound in 2023, showing a 110% increase.

#### Trends in MDR bacteria

**Figure 4.**
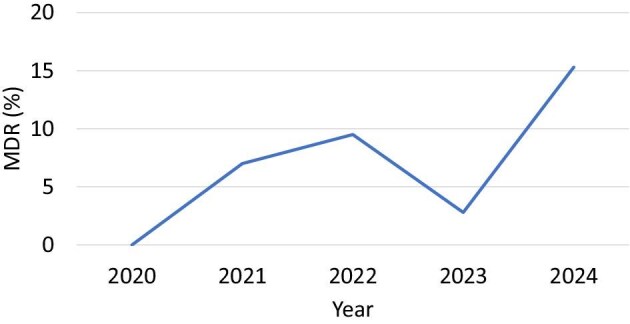
Trends of MDR bacteria over time. MDR: multidrug-resistant.

The studies also reported the prevalence of MDR strains, defined as resistance to ≥3 classes of antimicrobials. The temporal trends in MDR bacteria over time are as follows: in 2020, no MDR isolates were reported. By 2021, 25 MDR isolates had been documented, accounting for 7.0% of the total isolates. In 2022, the number of MDR isolates increased to 34, representing 9.5% of the total number of isolates. However, in 2023, a decline was observed, with only 10 MDR isolates reported, representing 2.8% of the total. Finally, in 2024, a significant rise was noted, with 55 MDR isolates recorded, comprising 15.3% of the total (Figure 4).

#### Key pathogens and their movements over time

**Figure 5.**
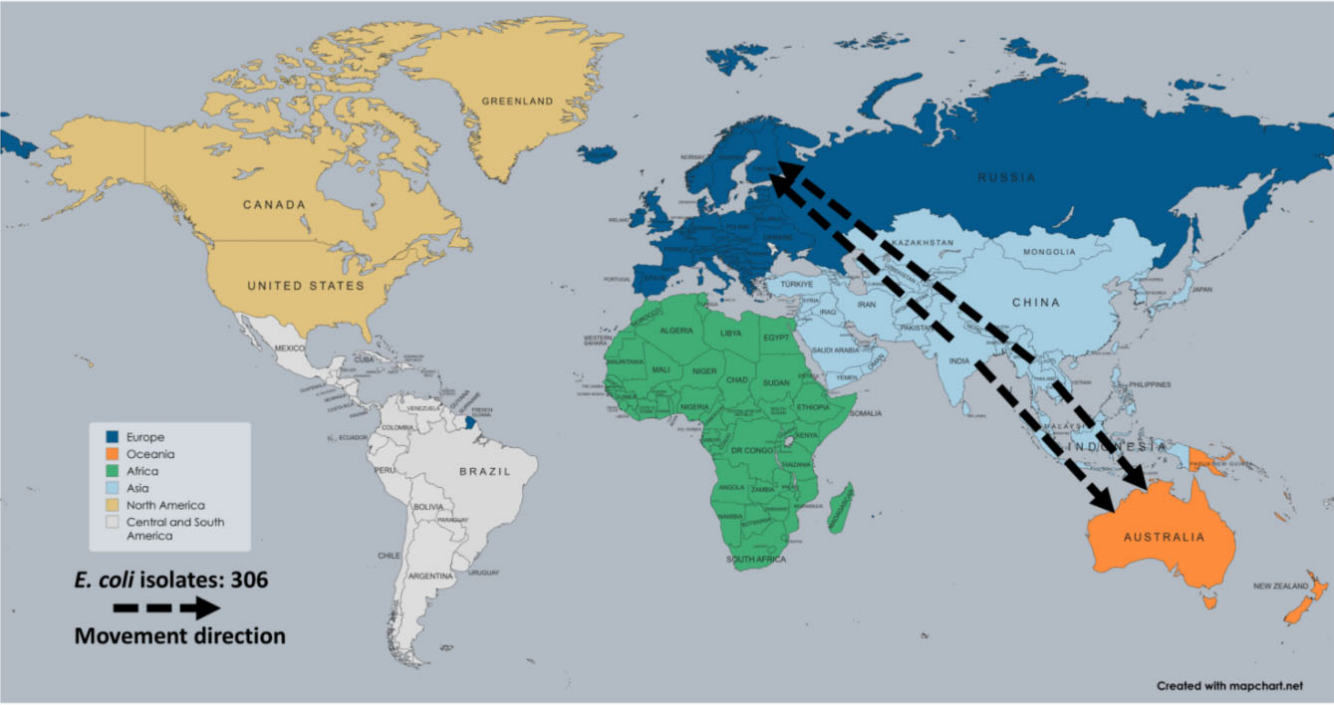
Travel-related antimicrobial resistance to *E. coli* transmission, 2020–2024.

The studies have primarily focused on key AMR pathogens and their movements over the years, with *E. coli* being the most commonly reported. In seven studies, 306 *E. coli* isolates were documented. The movement of AMR *E. coli* was predominantly observed from Southeast Asia, particularly India and Thailand, to Europe (Finland) and Oceania (Australia) (Figure 5). Notably, the number of travel-associated AMR *E. coli* cases has increased over the years, with the majority reported in 2022 and 2024.

**Figure 6.**
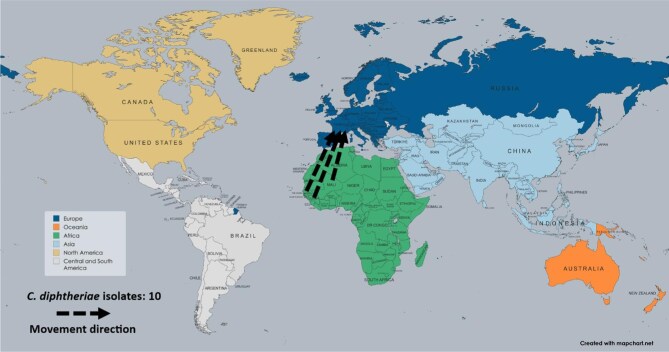
Travel-related antimicrobial resistance of *C. diphtheriae* transmission, 2020–2024.

*Corynebacterium diphtheriae* was reported in one study, which documented 10 AMR isolates originating from West Africa (Guinea, Mali, Senegal) that moved to France (Figure 6). This study highlighted the emergence of MDR *C. diphtheriae* strains in 2023.

**Figure 7.**
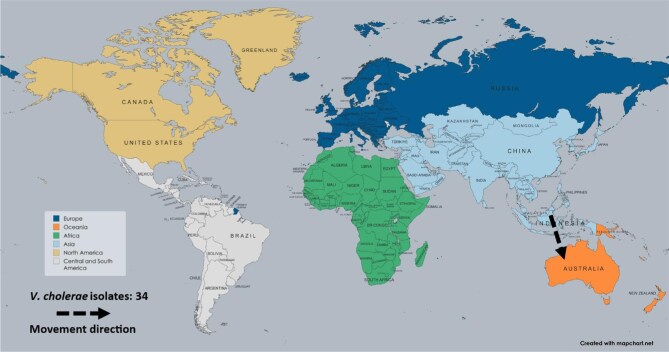
Travel-related antimicrobial resistance of *V. cholerae* transmissions, 2020–2024.

*Vibrio cholerae* was documented in one study, with 34 AMR isolates originating from Indonesia and moving to Australia in 2024 (Figure 7). These trends underscore the global dissemination of resistant bacteria facilitated by international travel, highlighting a notable increase in the number of reported cases and the prevalence of MDR strains.

#### Geographical trends

The data revealed distinct geographical patterns in the origins and destinations of travel-associated AMR bacteria. Southeast Asia emerged as a significant hotspot, accounting for most AMR isolates in 2022 and 2024. Africa, especially Zanzibar, was another notable source that contributed to the cases reported in 2023. A smaller number of AMR isolates were linked to travel to Latin America, specifically Peru, which was associated with the emergence of colistin resistance. Additionally, some AMR isolates were reported from unspecified or multiple regions, highlighting the global nature of this issue. These geographical trends underscore the need for targeted interventions and enhanced surveillance in high-risk regions, such as Southeast Asia and parts of Africa, to mitigate the cross-border dissemination of AMR.

### Healthcare exposure as a risk factor

The analysis identified healthcare exposure during travel, specifically among individuals who visited healthcare facilities such as hospitals or clinics, as a significant risk factor for acquiring AMR gut bacteria. This association was particularly evident for ESBL-producing *E. coli* (Table 1). Travellers who received medical care in certain regions, including Southeast Asia and parts of Africa, faced an elevated risk of colonisation with AMR bacteria. These areas may serve as reservoirs for resistant organisms, increasing the likelihood of acquisition in healthcare settings where infection control measures may be suboptimal. Consequently, such travellers may unknowingly carry and transmit these resistant strains upon return to their home countries, contributing to the global spread of AMR.

**Table 1.** Risk of healthcare exposure

Study	Bacteria	Origin	Destination	Outcome
Furuya-Kanamori et al.^18^	AMR *E. coli*	India, Thailand and Nepal	Australia	High rate of asymptomatic colonisation. Healthcare exposures during travel are significant for acquisition.
Kantele et al.^20^	ESBL-producing *E. coli*	Tropics	Finland	Travellers' diarrhoea is the most common clinical manifestation. Healthcare exposure during travel emphasised.
Moser et al.^21^	MDR *E. coli*	Zanzibar	Switzerland	Colonisation linked to interactions with local populations and healthcare settings.
Rondinaud et al.^22^	Enterobacterales carrying the mcr gene	Southeast Asia and Peru	France	Acquisition following travel. Healthcare exposure during travel.

AMR: antimicrobial-resistant; ESBL: Extended Spectrum Beta-Lactamase; MDR: multidrug-resistant.

## Discussion

Our systematic review revealed several worrying trends in the global movements and dissemination of travel-associated AMR bacteria. The analysis of the included studies highlighted the unidirectional flow of AMR bacteria, with certain regions, such as the Indian subcontinent, Southeast Asia and parts of Africa, serving as persistent hotspots for the origination and spread of these resistant microorganisms. These regions exhibited a high prevalence and diversity of resistant pathogens, which were then introduced to other parts of the world via international travel. This unidirectional pattern of AMR dissemination aligns with the broader global trends observed recently.^5,8^ It should be noted that the review by Bokhary et al.^5^ in 2021 also reported Asia as a significant reservoir for various AMR bacteria, indicating that the trend has not changed. The high burden of AMR in these regions is attributed to a combination of factors, including suboptimal antibiotic stewardship, weak infection control measures, poor sanitation and hygiene, as well as the presence of environmental reservoirs of resistance genes.^25,26^ The consistent identification of these regions as the primary sources of travel-associated AMR highlights the need for targeted interventions and collaborations to address the root causes of AMR in these hotspot areas. Strengthening antimicrobial stewardship, improving infection prevention and control practices in healthcare and community settings, as well as enhancing environmental surveillance and remediation efforts, are crucial steps in mitigating the generation and dissemination of resistant pathogens from these regions.

On the other hand, the limited data from Latin America and the Middle East represent a notable gap that deserves further exploration. One potential reason for the lack of representation from these regions could be the uneven distribution of research and surveillance efforts globally. Many low- and middle-income countries, particularly in Latin America and the Middle East, may have limited resources, infrastructure and technical capacity for robust AMR monitoring and reporting. This could result in an underestimation of the true burden of travel-associated AMR in these regions. Additionally, cultural, political and economic factors may influence the patterns of international travel and, consequently, the dissemination of resistant pathogens. For example, the political and economic ties between certain regions and travel flows may differ, which could impact the observed geographic trends. Future research should prioritize the expansion of AMR surveillance and reporting capabilities in Latin America and the Middle East, potentially through international collaborations and capacity-building initiatives.

We also observed an alarming diversification of resistant pathogens associated with international travel. While the initial focus was primarily on ESBL-producing *E. coli*, studies are now reporting an increasing occurrence of other concerning resistant organisms, such as carbapenem-resistant *Acinetobacter*, high-level ciprofloxacin-resistant *Salmonella* Typhi, MDR *Corynebacterium diphtheriae* and Enterobacterales carrying the colistin resistance gene mcr. The diversification of resistant microorganisms reflects the dynamic and evolving nature of the AMR challenge, which is outpacing our ability to effectively monitor and respond to these threats. The emergence and rapid dissemination of novel resistant pathogens, often facilitated by international travel, underscore the need for enhanced global surveillance and early warning systems.^27,28^ The WHO’s Global Antimicrobial Resistance and Use Surveillance System (GLASS) is a critical initiative in this regard, aiming to standardise data collection and reporting across countries.^29^ However, greater integration of travel-related AMR data into GLASS and similar platforms, such as the ECDC guidance on the prevention and control of CRE,^30^ could improve real-time monitoring and inform targeted interventions. Expanding the laboratory capacity and diagnostic infrastructure in low- and middle-income countries, where the burden of AMR is often the highest, is crucial to enabling the timely detection and characterisation of emerging resistant strains.^31,32^ Additionally, the development of rapid and reliable diagnostic tools that can be deployed in various settings, including points of entry and healthcare facilities, can improve the detection and tracking of these pathogens.

The role of travellers as vectors for the introduction and dissemination of AMR bacteria was consistently demonstrated, indicating that infections in travellers' home communities are often linked to travel-related transmission. This underscores the significant public health impact of international travel in driving the global spread of these pathogens. This finding aligns with the growing body of evidence that has recognised the critical role of human mobility in the global dissemination of AMR.^33,34^ Travellers, whether they are tourists, migrants, students or healthcare workers, can act as conduits for the introduction and spread of resistant microorganisms across borders. The increased frequency and volume of international travel, coupled with the growing prevalence of AMR in certain regions, have amplified this threat. Addressing the role of travellers in the transmission of AMR requires a multifaceted approach. Pretravel health consultations and education campaigns should emphasise the risks of acquiring resistant infections and the importance of preventive measures, such as hand hygiene, appropriate antibiotic use and prudent use of medical services during travel.^33,35^ Some national health agencies, such as the UK Health Security Agency^36^ and the US Centers for Disease Control and Prevention,^37^ have begun integrating AMR risk awareness into travel health guidelines, offering a model for broader adoption. Additionally, targeted screening and surveillance programmes for high-risk travellers, particularly those returning from regions with high AMR burdens, can also help identify and contain the spread of resistant microorganisms.

Our analysis of the included studies highlighted healthcare exposures during international travel as a significant risk factor for the acquisition of AMR gut bacteria, particularly Enterobacteriaceae. This finding underscores the critical role of healthcare settings in the global amplification and dissemination of resistant pathogens. Healthcare facilities in certain regions, such as the Indian subcontinent, Southeast Asia and parts of Africa, were identified as reservoirs for ESBL-producing *E. coli*. Travellers visiting these regions and accessing local healthcare services are at an increased risk of acquiring and subsequently introducing these resistant microorganisms into their home communities. Therefore, strengthening infection prevention and control measures in healthcare facilities in high-risk regions must be a priority. The WHO's Core IPC Components provide a useful framework for improving standards and reducing nosocomial transmission.^38^

Moreover, improving the availability and access to essential antimicrobials, as well as ensuring their appropriate use, can also help mitigate the selection and spread of resistant bacteria in these settings. Initiatives like the Access, Watch, and Reserve (AWaRe) antibiotic classification system, also promoted by the WHO, offer guidance for optimising antibiotic use and preserving last-resort agents.^39^ Additionally, promoting pretravel health consultations and educating travellers on the risks associated with healthcare exposures during travel can empower individuals to make informed decisions and take appropriate precautions. Targeted surveillance and screening programmes for travellers returning from regions with high AMR prevalence can also facilitate the early detection and containment of resistant infections.

### Limitations

Although this systematic review presents a valuable synthesis of the current evidence on the global movements and dissemination of travel-associated AMR bacteria, it is important to acknowledge the limitations of the available evidence and the potential implications for future research and policy considerations. The review was limited to studies published in English, which may have resulted in the exclusion of relevant studies published in other languages. The included studies predominantly focused on AMR dissemination from certain regions, such as Southeast Asia and Africa, whereas other potentially important regions (e.g. Middle East, Latin America) were under-represented. This uneven geographic distribution of the data may limit the generalizability of the findings and the ability to draw conclusions about global trends. Moreover, the included studies varied according to their methodologies, AMR reporting profiles and the level of detail reported. This heterogeneity may hinder the direct comparison and synthesis of findings across studies, potentially affecting the robustness of the conclusions. The reliance on published literature may underestimate the true burden of travel-associated AMR because cases may go undetected or unreported, particularly in resource-limited settings with limited surveillance and diagnostic capacities. The dissemination of AMR bacteria is influenced by a myriad of factors, including environmental, sociocultural and economic determinants, which may not be fully captured in the available literature. The literature search was confined to three major electronic databases, which may have led to the exclusion of studies not indexed in these sources. Despite these limitations, this systematic review offers a valuable synthesis of the current literature on the relationship between international travel and the acquisition of antimicrobial-resistant pathogens. The findings can inform future research, policy and interventions aimed at addressing the global challenge of travel-associated AMR.

## Conclusion

This systematic review revealed trends in the global movement and dissemination of travel-associated antimicrobial-resistant bacteria. The analysis highlights a unidirectional flow of AMR bacteria, with Southeast Asia and Africa serving as persistent hotspots for the origination and spread of these resistant microorganisms. The diversification of resistant pathogens, the role of travellers as vectors and the significant risk associated with healthcare exposures during travel underscore the escalating public health challenge posed by the international spread of AMR. To address this threat, a multifaceted approach is required, involving enhanced global surveillance and early warning systems, improved antimicrobial stewardship and infection prevention and control measures, targeted traveller education and interventions, as well as strengthened global collaboration and policy harmonisation. Sustained efforts in these areas, coupled with expanded research to address knowledge gaps, are essential to combat the global dissemination of travel-associated AMR and safeguarding public health worldwide.

## Recommendations and future directions

Based on the findings of this systematic review, several key recommendations and future research directions can be proposed to address the challenge of travel-associated AMR. These recommendations align with existing policy frameworks and guidelines, such as the WHO's Global Action Plan on Antimicrobial Resistance and the ECDC's guidance on the prevention and control of CRE.

Short-term:

- Develop and implement reliable, rapid and portable diagnostic tools that can be deployed at entry points and in healthcare facilities to screen high-risk travellers.
- Establish integrated cross-border surveillance networks to track the movements and dissemination of AMR bacteria globally.

Long-term:

- Expanding the laboratory capacity and diagnostic infrastructure in low- and middle-income countries to enable the timely detection and characterisation of emerging resistant pathogens.

## Supplementary Material

ihaf071_Supplemental_File

## Data Availability

All supporting data are presented in the manuscript and supplementary files.

## References

[1] ANTIMICROBIAL RESISTANCE Global Report on Surveillance (2014). https://iris.who.int/bitstream/handle/10665/112642/9789241564748_eng.pdf?sequence=1 [accessed 23 May 2025].

[2] Boolchandani M, Blake KS, Tilley DH et al. Impact of international travel and diarrhea on gut microbiome and resistome dynamics. Nat Commun. 2022;13(1):7485. 36470885 10.1038/s41467-022-34862-w PMC9722912

[3] Dallman TJ, Neuert S, Fernandez Turienzo C et al. Prevalence and persistence of antibiotic resistance determinants in the gut of travelers returning to the United Kingdom is associated with colonization by pathogenic Escherichia coli. Microbiol Spectr. 2023;11(4):e0518522. 37255437 10.1128/spectrum.05185-22 PMC10433802

[4] International tourism growth continues to outpace the global economy | UN Tourism. Available from: https://www.unwto.org/international-tourism-growth-continues-to-outpace-the-economy [accessed 5 January 2025].

[5] Bokhary H, Pangesti KNA, Rashid H et al. Travel-related antimicrobial resistance: a systematic review. Trop Med Infect Dis. 2021;6(1):11. 33467065 10.3390/tropicalmed6010011 PMC7838817

[6] Poudel AN, Zhu S, Cooper N et al. The economic burden of antibiotic resistance: A systematic review and meta-analysis. PLoS One. 2023;18(5):e0285170. 37155660 10.1371/journal.pone.0285170 PMC10166566

[7] Sparrow A, Smith-Torino M, Shamamba SM et al. A risk management approach to global pandemics of infectious disease and anti-microbial resistance. Trop Med Infect Dis. 2024;9(11):280. 39591286 10.3390/tropicalmed9110280 PMC11598814

[8] Voor In ’T, Holt AF, Mourik K et al. Acquisition of multidrug-resistant enterobacterales during international travel: a systematic review of clinical and microbiological characteristics and meta-analyses of risk factors. Antimicrob Resist Infect Control. 2020;9(1):71. 10.1186/s13756-020-00733-6 32434591 PMC7237615

[9] Kuenzli E, Jaeger VK, Frei R et al. High colonization rates of extended-spectrum β-lactamase (ESBL)-producing Escherichia coli in Swiss travellers to South Asia- a prospective observational multicentre cohort study looking at epidemiology, microbiology and risk factors. BMC Infect Dis. 2014;14(528):1471–2334. 10.1186/1471-2334-14-528 PMC4262238 25270732

[10] Paltansing S, Vlot JA, Kraakman MEM et al. Extended-spectrum β-lactamase–producing Enterobacteriaceae among travelers from the Netherlands. Emerg Infect Dis. 2013;19(8):1206. 23885972 10.3201/eid.1908.130257 PMC3739527

[11] Carbapenem-resistant Enterobacteriaceae. 2016. https://www.ecdc.europa.eu/sites/default/files/media/en/publications/Publications/carbapenem-resistant-enterobacteriaceae-risk-assessment-april-2016.pdf [accessed 23 May 2025].

[12] Page MJ, McKenzie JE, Bossuyt PM et al. The PRISMA 2020 statement: an updated guideline for reporting systematic reviews. BMJ. 2021;372:n71. 33782057 10.1136/bmj.n71 PMC8005924

[13] Ouzzani M, Hammady H, Fedorowicz Z et al. Rayyan—A web and mobile app for systematic reviews. Syst Rev. 2016;5(1):210. 27919275 10.1186/s13643-016-0384-4 PMC5139140

[14] Wells GA, Wells G, Shea B et al. The Newcastle-Ottawa Scale (NOS) for assessing the quality of nonrandomised studies in meta-analyses. 2014. https://www.semanticscholar.org/paper/The-Newcastle-Ottawa-Scale-(NOS)-for-Assessing-the-Wells-Wells/c293fb316b6176154c3fdbb8340a107d9c8c82bf [accessed 23 May 2025].

[15] Bote L, Taylor-Brown A, Maes M et al. Surveillance of travel-associated isolates elucidates the diversity of non-pandemic Vibrio cholerae. Microb Genom. 2024;10(10):001307. 10.1099/mgen.0.001307 39412871 PMC11900828

[16] Brémont S, Passet V, Hennart M et al. Multidrug-resistant Corynebacterium diphtheriae in people with travel history from West Africa to France, March to September 2023. Euro Surveill. 2023;28(46):2300615. 10.2807/1560-7917.ES.2023.28.46.2300615 37971662 PMC10655204

[17] Buchek G, Mende K, Telu K et al. Travel-associated multidrug-resistant organism acquisition and risk factors among US military personnel. J Travel Med. 2021;28(3):taab028. 10.1093/jtm/taab028 33675647 PMC8045176

[18] Furuya-Kanamori L, Mills DJ, Trembizki E et al. High rate of asymptomatic colonization with antimicrobial-resistant Escherichia coli in Australian returned travellers. J Travel Med. 2022;29(1):taab141. 10.1093/jtm/taab141 34494119

[19] Kantele A, Lääveri T. Extended-spectrum beta-lactamase-producing strains among diarrhoeagenic Escherichia coli-prospective traveller study with literature review. J Travel Med. 2022;29(1):taab042. 10.1093/jtm/taab042 33834207 PMC8763120

[20] Kantele A, Lääveri T, Mero S et al. Despite predominance of uropathogenic/extraintestinal pathotypes among travel-acquired extended-spectrum β-lactamase-producing Escherichia coli, the most commonly associated clinical manifestation is travelers’ diarrhea. Clin Infect Dis. 2020;70(2):210–8. 31034006 10.1093/cid/ciz182 PMC6938974

[21] Moser AI, Kuenzli E, Büdel T et al. Travellers returning from the island of Zanzibar colonized with MDR Escherichia coli strains: assessing the impact of local people and other sources. J Antimicrob Chemother. 2021;76(2):330–7. 33257991 10.1093/jac/dkaa457

[22] Rondinaud E, Clermont O, Petitjean M et al. Acquisition of enterobacterales carrying the colistin resistance gene mcr following travel to the tropics. J Travel Med. 2023;30(1):taac141. 10.1093/jtm/taac141 36444951

[23] Tun HM, Cowling BJ, Bruzzone R et al. Acquisition of antimicrobial resistance after travel to resource-limited countries: a multi-layer metagenomic epidemiological study (abridged secondary publication). Hong Kong Med J. 2024;30:16–19. Available from: www.hkmj.org 38962917

[24] Shin E, Park J, Jeong HJ et al. Emerging high-level ciprofloxacin-resistant Salmonella enterica serovar typhi haplotype H58 in travelers returning to the Republic of Korea from India. PLoS Negl Trop Dis. 2021;15(3):e0009170. 10.1371/journal.pntd.0009170 PMC7987170 33651791

[25] Gulumbe BH, Haruna UA, Almazan J et al. Combating the menace of antimicrobial resistance in Africa: a review on stewardship, surveillance and diagnostic strategies. Biol Proced Online. 2022;24(1):1–13. 36424530 10.1186/s12575-022-00182-y PMC9685880

[26] Moyo P, Moyo E, Mangoya D et al. Prevention of antimicrobial resistance in sub-Saharan Africa: What has worked? What still needs to be done? J Infect Public Health. 2023;16(4):632–9. 36870230 10.1016/j.jiph.2023.02.020

[27] Creppage KE, Gallaway MS, Russell DA et al. Global Emerging Infections Surveillance Program contributions to pandemic preparedness and response. Emerg Infect Dis. 2024;30(14):9–12. 39530771 10.3201/eid3014.240305 PMC11559581

[28] Iera J, Iera J, Isonne C et al. National early warning systems for emerging AMR in high-income countries: a systematic review. Eur J Public Health. 2024;34(Supplement_3):ckae144.136. Available from: 10.1093/eurpub/ckae144.136

[29] Global Antimicrobial resistance and Use surveillance system (GLASS). Available from: https://www.who.int/initiatives/glass [accessed 23 May 2025].

[30] Carbapenem-resistant enterobacterales (CRE). Available from: https://www.ecdc.europa.eu/en/publications-data/directory-guidance-prevention-and-control/prevention-and-control-infections-1 [accessed 23 May 2025].

[31] Yamba K, Chizimu JY, Mudenda S et al. Assessment of antimicrobial resistance laboratory-based surveillance capacity of hospitals in Zambia: findings and implications for system strengthening. J Hosp Infect. 2024;148:129–37. 38621513 10.1016/j.jhin.2024.03.014 PMC11171463

[32] Antimicrobial resistance Diagnostic Initiative strengthening bacteriology and mycology diagnostic capacity, laboratory systems and service delivery. 2023. Available from: https://www.who.int/ [accessed 12 January 2025].

[33] Sridhar S, Turbett SE, Harris JB et al. Antimicrobial-resistant bacteria in international travelers. Curr Opin Infect Dis. 2021;34(5):423. 34267046 10.1097/QCO.0000000000000751 PMC8452315

[34] Desai AN, Mohareb AM, Hauser N et al. Antimicrobial resistance and human mobility. Infect Drug Resist. 2022;15:127. 35046676 10.2147/IDR.S305078 PMC8763254

[35] Arieti F, Savoldi A, Rejendran NB et al. The antimicrobial resistance travel tool, an interactive evidence-based educational tool to limit antimicrobial resistance spread. J Travel Med. 2022;29(4):1–13. 10.1093/jtm/taac045 PMC9282094 35348740

[36] Confronting antimicrobial resistance 2024 to 2029–GOV.UK. Available from: https://www.gov.uk/government/publications/uk-5-year-action-plan-for-antimicrobial-resistance-2024-to-2029/confronting-antimicrobial-resistance-2024-to-2029 [accessed 23 May 2025].

[37] About Antimicrobial Resistance Investments & Action | Antimicrobial Resistance | CDC. Available from: https://www.cdc.gov/antimicrobial-resistance/programs/AR-investments.html [accessed 23 May 2025].

[38] Core competencies for infection prevention and control professionals. 2020. Available from: http://apps.who.int/bookorders [accessed 23 May 2025].

[39] AWaRe classification of antibiotics for evaluation and monitoring of use, 2023. Available from: https://www.who.int/publications/i/item/WHO-MHP-HPS-EML-2023.04 [accessed 23 May 2025].

