# Supplementary material for: Global trends in travel-related antimicrobial resistance: a systematic review, 2020–2024
Source: Int Health. 2025 Oct 29;18(3):395–405. doi: 10.1093/inthealth/ihaf071 (PMC13154825; doi:10.1093/inthealth/ihaf071)
Supplement: ihaf071_Supplemental_File [file ihaf071_supplemental_file.docx]

Table S1. Study characteristics

| Publication year | First Author's last name | Title | Listed reduced sensitivity species | Reported reduced sensitivity or resistant antimicrobials | Traveling from | Traveling to | Assessment score |
| --- | --- | --- | --- | --- | --- | --- | --- |
| 2024 | Bote | Surveillance of travel-associated isolates elucidates the diversity of non-pandemic Vibrio cholerae | Vibrio cholerae | tetracycline, doxycycline, ciprofloxacin | Indonesia | Australia | 7 |
| 2023 | Bremont | Multidrug-resistant Corynebacterium diphtheriae in people with travel history from West Africa to France, March to September 2023 | Corynebacterium diphtheriae | gentamicin, tetracycline, benzylpenicillin, sulphonamide, trimethoprim, ciprofloxacin, azithromycin | Guinea, Mali, Senegal, Niger, Nigeria, Central African Republic | France | 6 |
| 2021 | Buchek | Travel-associated multidrug-resistant organism acquisition and risk factors among US military personnel | Escherichia coli, Acinetobacter, Aeromonas, Citrobacter, Enterobacter, klebsiella, Kluyvera, Morganella, Pantoea, proteus, Pseudomonas, Stenotrophomonas | cefazolin, trimethoprim-sulfamethoxazole, tetracycline, ciprofloxacin, levofloxacin, tobramycin, gentamicin, nitrofurantoin | Mexico, the Caribbean, Central America, Asia, Africa, South America | USA | 6 |
| 2021 | Furuya-Kanamori | High rate of asymptomatic colonization with antimicrobial-resistant Escherichia coli in Australian returned travellers | Escherichia coli | amoxicillin, cefazolin, ceftriaxone, ciprofloxacin, cotrimoxazole, amoxicillin-clavulanate, cefepime, gentamicin, piperacillin-tazobactam, tobramycin | India, Thailand, Nepal | Australia | 8 |
| 2022 | Kantele | Extended-spectrum beta-lactamase-producing strains among diarrhoeagenic Escherichia coli: a prospective traveller study with literature review | Escherichia coli | ciprofloxacin, azithromycin, rifaximin | South Asia, Southeast Asia | Finland | 7 |
| 2020 | Kantele | Despite Predominance of Uropathogenic/Extraintestinal Pathotypes Among Travel-acquired Extended-spectrum beta-lactamase-producing Escherichia coli, the Most Commonly Associated Clinical Manifestation Is Travelers' Diarrhea | Escherichia coli | tobramycin, Ciprofloxacin, Cotrimoxazole | Tropics | Finland | 8 |
| 2020 | Moser | Travelers returning from the island of Zanzibar colonized with MDR Escherichia coli strains: assessing the impact of local people and other sources | Escherichia coli | colistin, cefuroxime | Island of Zanzibar | Switzerland | 7 |
| 2023 | Rondinaud | Acquisition of Enterobacterales carrying the colistin resistance gene mcr following travel to the tropics | Escherichia coli, Escherichia albertii, Escherichia cloacae | colistin | Southeast Asia, Peru | France | 7 |
| 2024 | Tun | Acquisition of antimicrobial resistance after travel to resource-limited countries: a multi-layer metagenomic epidemiological study (abridged secondary publication) | Escherichia coli | amoxicillin, ceftazidime, cefepime, gentamycin, tobramycin, azithromycin, tetracycline, ciprofloxacin, norfloxacin, trimethoprim-sulfamethoxazole, chloramphenicol |  | Hong kong | 7 |
| 2021 | Shin | Emerging high-level ciprofloxacin-resistant Salmonella enterica serovar typhi haplotype H58 in travellers returning to the Republic of Korea from India | Salmonella typhi | Ciprofloxacin | India | Korea | 8 |
|  |  |  |  |  |  |  |  |

Table S2. Newcastle-Ottawa Scale (NOS)

|  | Selection | | | | Comparability | Outcome | | |  |  | |
| --- | --- | --- | --- | --- | --- | --- | --- | --- | --- | --- | --- |
| First Author's last name | Representativeness of the Exposed Cohort | Selection of the Non-Exposed Cohort | Ascertainment of Exposure | Demonstration that Outcome of Interest Was Not Present at Start of Study | Comparability of Cohorts on the Basis of the Design or Analysis | Assessment of Outcome | Was Follow-Up Long Enough for Outcomes to Occur | Adequacy of Follow-Up of Cohorts | Total Score | Quality | |
| Bote | * | * | * | - | * | * | * | * | 7 | good | |
| Bremont | * | * | * | * | - | * | - | * | 6 | fair | |
| Buchek | * | * | * | * | - | * | - | * | 6 | fair | |
| Furuya-Kanamori | * | * | * | * | * | * | * | * | 8 | good | |
| Kantele | * | * | * | * | - | * | * | * | 7 | good | |
| Kantele | * | * | * | * | * | * | * | * | 8 | good | |
| Moser | * | * | * | * | - | * | * | * | 7 | good | |
| Rondinaud | * | * | * | * | - | * | * | * | 7 | good | |
| Tun | * | * | * | * | - | * | * | * | 7 | good | |
| Shin | * | * | * | * | * | * | * | * | 8 | good | |
| “*” indicate that the article meets the criteria mentioned above; “-” indicate that the article does not meet the criteria mentioned above. | | | | | | | | | | |  |

Table S3. Top originating regions for AMR organisms (source).

|  |  |  |  |  |  |  |  |
| --- | --- | --- | --- | --- | --- | --- | --- |

|  | | | | | | | |
| --- | --- | --- | --- | --- | --- | --- | --- |
| **#** | **Region** | **Isolates** | **Studies** | **##** | **Top Locations** | **Isolates** | **Studies** |
| 1 | Southeast Asia | 58 | 5 | 1 | Indonesia | 34 | 1 |
|  | Asia | 102 | 2 | 2 | India | 26 | 2 |
|  |  |  |  | 3 | Thailand | 4 | 1 |
|  |  |  |  | 4 | Nepal | 4 | 1 |
|  |  |  |  | 5 | Unspecified* | 92 | 2 |
| 2 | Africa | 70 | 6 | 1 | Guinea | 3 | 1 |
|  |  |  |  | 2 | Mali | 3 | 1 |
|  |  |  |  | 3 | Senegal | 2 | 1 |
|  |  |  |  | 4 | Nigeria | 1 | 1 |
|  |  |  |  | 5 | Central Africa Republic | 1 | 1 |
|  |  |  |  | 6 | Zanzibar | 60 | 1 |
| 3 | Others | 134 | 5 | 1 | Unspecified* | 134 | 5 |
| 4 | Latin America | 4 | 1 | 1 | Peru | 4 | 1 |
| *No mention of a specific travel source | | | |  |  |  |  |

Table S4. Top destination regions for AMR organisms

|  |  |  |  |  | |  | |  | |  | |
| --- | --- | --- | --- | --- | --- | --- | --- | --- | --- | --- | --- |
| # | **Region** | **Isolates** | **Studies** | **##** | **Top Locations** | | **Isolates** | | **Studies** | |  |
| 1 | Oceania | 68 | 2 | 1 | Australia | | 68 | | 2 | |  |
|  |  |  |  |  |  | |  | |  | |  |
| 2 | Europe | 207 | 5 | 1 | France | | 38 | | 2 | |  |
|  |  |  |  | 2 | Finland | | 109 | | 2 | |  |
|  |  |  |  | 3 | Switzerland | | 60 | | 1 | |  |
|  |  |  |  |  |  | |  | |  | |  |
| 3 | North America | 25 | 1 | 1 | USA | | 25 | | 1 | |  |
|  |  |  |  |  |  | |  | |  | |  |
| 4 | East Asia | 68 | 2 | 1 | Hong-Kong | | 60 | | 1 | |  |
|  |  |  |  | 2 | South Korea | | 8 | | 1 | |  |
| *No mention of a specific travel source | | | | | | |  |  |  |  |  |
|  |  |  |  |  | |  | |  | |  | |

Table S5. Number of studies and isolates for the species that were documented in the analyzed studies.

|  |  |  |  |
| --- | --- | --- | --- |
|  |  | Number of documented travelling AMR | |
| # | species | studies | isolates |
| 1 | Citrobacter freundi | 1 | 5 |
| 2 | Corynebacterium diphtheriae | 1 | 10 |
| 3 | Escherichia albertii | 1 | 1 |
| 4 | Escherichia cloacae | 1 | 3 |
| 5 | Escherichia coli | 7 | 306 |
| 6 | Vibrio cholerae | 1 | 34 |
| 7 | Salmonella typhi | 1 | 8 |

Table S6. Antimicrobials categories that were included in the analysis.

|  |
| --- |

| \| Antibiotic class \| \| mentioned antimicrobial names \| \| \|  \|  \|  \|  \| \| --- \| --- \| --- \| --- \| --- \| --- \| --- \| --- \| --- \| \| Beta Lactams \| penicillins \| benzylpenicillin, amoxicillin, amoxicillin/clavulanate, piperacillin/tazobactam. \| \| \| \| \| \| \| \| cephalosporins \| cefazolin, ceftriaxone, cefepime, cefuroxime, ceftazidime. \| \| \| \|  \|  \|  \| \| macrolides and lincosamides \| \| azithromycin, \|  \|  \|  \|  \|  \|  \| \| quinolones \| \| ciprofloxacin, levofloxacin, norfloxacin, nalidixic acid, \| \| \| \|  \|  \|  \| \| sulfonamides and trimethoprim \| \| sulphonamide, trimethoprim, trimethoprim-sulfamethoxazole, \| \| \| \| \|  \|  \| \| tetracyclines \| \| tetracycline, doxycycline, \| \|  \|  \|  \|  \|  \| \| aminoglycosides \| \| gentamicin, tobramycin, \| \|  \|  \|  \|  \|  \| \| amphenicol \| \| chloramphenicol, \|  \|  \|  \|  \|  \|  \| \| Others/not classified \| \| nitrofurantoin, colistin \| \|  \|  \|  \|  \|  \| |
| --- | --- | --- | --- | --- | --- | --- | --- | --- | --- | --- | --- | --- | --- | --- | --- | --- | --- | --- | --- | --- | --- | --- | --- | --- | --- | --- | --- | --- | --- | --- | --- | --- | --- | --- | --- | --- | --- | --- | --- | --- | --- | --- | --- | --- | --- | --- | --- | --- | --- | --- | --- | --- | --- | --- | --- | --- | --- | --- | --- | --- | --- | --- | --- | --- | --- | --- | --- | --- | --- | --- | --- | --- | --- | --- | --- | --- | --- | --- | --- | --- | --- | --- | --- | --- | --- | --- | --- | --- | --- |

Table S7. Numbers of travel-related AMR isolates documented in 10 studies, from 2020 to 2024, categorized by source.

| All organisms | | | As | | | | | | SEas | | | | | | Af | | | | | | Lam | | | | | | Others | | | | | |
| --- | --- | --- | --- | --- | --- | --- | --- | --- | --- | --- | --- | --- | --- | --- | --- | --- | --- | --- | --- | --- | --- | --- | --- | --- | --- | --- | --- | --- | --- | --- | --- | --- |
| Antimicrobial  resistance component  (Number of isolates) | | 2020 | | 2021 | 2022 | 2023 | 2024 | Total | 2020 | 2021 | 2022 | 2023 | 2024 | Total | 2020 | 2021 | 2022 | 2023 | 2024 | Total | 2020 | 2021 | 2022 | 2023 | 2024 | Total | 2020 | 2021 | 2022 | 2023 | 2024 | Total |
|  | Any AMR | 0 | | 0 | 0 | 0 | 0 | **0** | 0 | 0 | 0 | 0 | 0 | **0** | 0 | 0 | 0 | 0 | 0 | **0** | 0 | 0 | 0 | 0 | 0 | **0** | 0 | 0 | 0 | 0 | 0 | **0** |
| Beta-Lactams | All (404) |  | | 0 | 0 | 0 | 0 | **0** | 0 | 0 | 0 | 0 | 0 | **0** | 0 | 0 | 0 | 0 | 0 | **0** | 0 | 0 | 0 | 0 | 0 | **0** | 0 | 0 | 0 | 0 | 0 | **0** |
|  | Penicillin (167) | 0 | | 0 | 102 | 0 | 55 | **157** | 0 | 0 | 0 | 0 | 0 | **0** | 0 | 0 | 0 | 10 | 0 | **10** | 0 | 0 | 0 | 0 | 0 | **0** | 0 | 0 | 0 | 0 | 0 | **0** |
|  | Carbapenems (0) | 0 | | 0 |  | 0 |  | **0** | 0 | 0 | 0 | 0 | 0 | **0** | 0 | 0 | 0 | 0 | 0 | **0** | 0 | 0 | 0 | 0 | 0 | **0** | 0 | 0 | 0 | 0 | 0 | **0** |
|  | Cephalosporins (237) | 0 | | 0 | 102 | 0 | 110 | **212** | 0 | 0 | 0 | 0 | 0 | **0** | 0 | 0 | 0 | 0 | 0 | **0** | 0 | 0 | 0 | 0 | 0 | **0** | 0 | 25 | 0 | 0 | 0 | **25** |
| Macrolides & Licosamide (76) | | 0 | | 8 |  | 0 | 55 | **63** | 0 | 0 | 0 | 0 | 0 | **0** | 0 | 0 | 0 | 0 | 0 | **0** | 0 | 0 | 0 | 0 | 0 | **0** | 0 | 0 | 13 | 0 | 0 | **13** |
| Quinolones (345) | | 0 | | 8 | 34 | 0 | 110 | **152** | 0 | 0 | 0 | 0 | 34 | **34** | 0 | 0 | 0 | 0 | 0 | **0** | 0 | 0 | 0 | 0 | 0 | **0** | 96 | 50 | 13 | 0 | 0 | **159** |
| Sulfonamides & Trimethoprim (220) | | 0 | | 0 | 34 | 0 | 55 | **89** | 0 | 0 | 0 | 0 | 0 | **0** | 0 | 0 | 0 | 10 | 0 | **10** | 0 | 0 | 0 | 0 | 0 | **0** | 96 | 25 | 0 | 0 | 0 | **121** |
| Tetracycline (171) | | 0 | | 0 |  | 0 | 55 | **55** | 0 | 0 | 0 | 0 | 68 | **68** | 0 | 0 | 0 | 10 | 0 | **10** | 0 | 0 | 0 | 0 | 0 | **0** |  | 25 | 13 | 0 | 0 | **38** |
| Aminoglycosides (367) | | 0 | | 8 | 68 | 0 | 110 | **186** | 0 | 0 | 0 | 0 | 0 | **0** | 60 | 0 | 0 | 10 | 0 | **70** | 0 | 0 | 0 | 0 | 0 | **0** | 96 | 15 | 0 | 0 | 0 | **111** |
| Amphicol (55) | | 0 | | 0 | 0 | 0 | 55 | **55** | 0 | 0 | 0 | 0 | 0 | **0** | 0 | 0 | 0 | 0 | 0 | **0** | 0 | 0 | 0 | 0 | 0 | **0** | 0 | 0 | 0 | 0 | 0 | **0** |
| Others (107) | | 0 | | 0 | 0 | 0 | 0 | **0** | 0 | 0 | 0 | 18 | 0 | **18** | 60 | 0 | 0 | 0 | 0 | **60** | 0 | 0 | 0 | 4 | 0 | **4** | 0 | 25 | 0 | 0 | 0 | **25** |
| MDR | | 0 | | 0 | 34 | 0 | 55 | **89** | 0 | 0 | 0 | 0 | 0 | **0** | 0 | 0 | 0 | 10 | 0 | **10** | 0 | 0 | 0 | 0 | 0 | **0** | 0 | 25 | 0 | 0 | 0 | **25** |

MDR, multidrug resistant organisms; organisms documented as multidrug resistant or resistant to three or more classes of antimicrobials**; Af:** Africa, includes isolates originated from Africa ; **As**, Asia, includes isolates originated from Asia but not specified from which country or area ; **SEas**: Southeast Asia; **Lam**: Latin America; **Other**: unspecified or multiple regions were documented.

Table S8. Search terms used for each database

| Database |  |  |
| --- | --- | --- |
| PubMed | Search results | Filter |
| ( "travel" OR "pilgrim*" OR "Hajj" OR "Hadj" OR "Haj" OR "Olympic" OR "overseas student" OR "international student" OR "immigrant" OR "world cup" OR "mass gathering" OR "crowding" OR "tourism" OR "travel medicine" OR "holiday" ) AND ( "drug resistance" OR "antimicrobial resistan*" ) | 1180 | January 2020 and December 2024 |
|  |  |  |
| Scopus |  |  |
| ( "travel" OR "pilgrim*" OR "Hajj" OR "Hadj" OR "Haj" OR "Olympic" OR "overseas student" OR "international student" OR "immigrant" OR "world cup" OR "mass gathering" OR "crowding" OR "tourism" OR "travel medicine" OR "holiday" ) AND ( "drug resistance" OR "antimicrobial resistan*" ) | 558 | January 2020 and December 2024 |
|  |  |  |
| Web of Science |  |  |
| TS=("travel" OR "pilgrim*" OR "Hajj" OR "Hadj" OR "Haj" OR "Olympic" OR "overseas student" OR "international student" OR "immigrant" OR "world cup" OR "mass gathering" OR "crowding" OR "tourism" OR "travel medicine" OR "holiday") AND TS=("drug resistance" OR "antimicrobial resistan*") | 338 | January 2020 and December 2024 |
| Manuel search in Google scholar and reference list | 10 | January 2020 and December 2024 |
